# Yield Stability of Soybean Variety Morkhor 60 in Integrated Rotation Systems of Northeastern Thailand

**DOI:** 10.3390/plants14162503

**Published:** 2025-08-12

**Authors:** Adisak Taiyawong, Tidarat Monkham, Jirawat Sanitchon, Sukanlaya Choenkwan, Sittipong Srisawangwong, Jamnan Khodphuwiang, Suntit Reewarabundit, Sompong Chankaew

**Affiliations:** 1 Department of Agronomy, Faculty of Agriculture, Khon Kaen University, Mueang District, Khon Kaen 40002, Thailand; adisak_ta@kkumail.com (A.T.); tidamo@kku.ac.th (T.M.); jirawat@kku.ac.th (J.S.); 2 Department of Agricultural Extension and Development, Faculty of Agriculture, Khon Kaen University, Mueang District, Khon Kaen 40002, Thailand; sukanl@kku.ac.th; 3 Khon Kaen Seed Research and Development Center, Tha-Phra Sub-District, Mueang District, Khon Kaen 40260, Thailand; sittipong.doa@gmail.com; 4 Mitr Phol Innovation & Research Center, 399 Moo 1 Khok Sa-At Sub-District, Phu Khiao District, Chaiyaphum 36110, Thailand; jamnank@mitrphol.com (J.K.); suntitr@mitrphol.com (S.R.)

**Keywords:** seed shortage, cropping system, genotype–environment interaction, yield stability, AMMI analysis, GGE biplot, agricultural sustainability, Thailand agriculture

## Abstract

Soybean (*Glycine max* (L.) Merrill) is globally valued for protein, oil, and biofuel applications. Thailand imports 99.8% of its soybean consumption due to declining cultivation areas. Northeastern Thailand presents substantial potential for expanding soybean production through integrated seed rotation systems in post-sugarcane (upland) and post-rice (lowland) environments. This study evaluated the newly developed ‘Morkhor 60’ soybean variety compared to three commercial varieties (SJ 5, 223*Lh-85, and CM 60) across thirteen diverse environments in Northeastern Thailand during 2022–2023. Field experiments employed a randomized complete block design with four replications per site. The ‘Morkhor 60’ demonstrated favorable yield stability and competitive performance across most environments. The variety showed broad adaptability across soil types (sandy loam to clay) and seasonal conditions (rainy and dry seasons) with minimal genotype-by-environment interactions. Chemical analysis revealed favorable protein content (39.63%) and oil content (14.66%). These findings support the cultivation of ‘Morkhor 60’ in integrated seed rotation systems, offering a viable strategy to reduce national soybean seed shortages while enhancing domestic production and agricultural sustainability.

## 1. Introduction

Soybean (*Glycine max* L. Merrill) ranks among the world’s most important legume crops, serving as a primary source of high-quality plant protein and oil for human consumption, livestock feed, and industrial applications, including biofuel production [1]. As the world’s largest source of animal protein feed and the second largest source of vegetable oil, soybeans comprise more than 10% of global agricultural trade value [2]. However, global soybean markets face unprecedented challenges, with production forecasts declining by over 5.1 million tons, creating significant supply pressures worldwide [3]. Thailand imports approximately 3.2 million tons of soybeans annually, with 88% sourced from Brazil and 10% from the United States, valued at approximately 1.7 billion USD [4]. This heavy dependence on imports, representing 99.8% of domestic consumption, highlights the urgent need to enhance domestic soybean production for food security and economic sustainability.

Soybeans were considered a minor crop in Thailand compared to major crops like rice, sugarcane, and cassava. The critical gap between domestic production capacity and consumption demands necessitates innovative approaches to increase local soybean cultivation without competing with established major crops. Strategic integration of soybeans into existing cropping systems through rotation offers a promising pathway to increase production while maximizing land use efficiency. Crop rotation systems featuring soybeans provide exceptional agronomic and economic benefits through biological nitrogen fixation (BNF), a symbiotic process between soybeans and *Bradyrhizobium japonicum* bacteria. Legumes like soybeans can fix atmospheric nitrogen and store it in root nodules, providing 30–40 kg of N_2_ for every ton of shoot dry matter produced [5]. Research demonstrates that rotating crops such as corn and soybeans increases yields by over 20% while reducing nitrous oxide emissions by 35%, highlighting productivity and environmental benefits [6].

However, the primary constraint limiting soybean area expansion in Thailand is acute seed shortage, compounded by the limited storage life of oilseed crop seeds and their requirement for seasonal replanting due to declining germination rates [7]. This biological limitation necessitates effective seed rotation systems between seasons and regions. Previously, Thailand implemented seed rotation from northern regions during the rainy season to northeastern areas during the dry season, but environmental incompatibility often compromised variety performance [8].

The success of seed rotation systems fundamentally depends on identifying soybean varieties that exhibit consistent performance across contrasting wet and dry environmental conditions. Varieties with extensive genotype-by-environment interactions cannot sustain effective seed rotation between complementary production windows, making identifying stable, high-yielding varieties critical for establishing viable rotation systems. Soybeans exhibit high sensitivity to environmental factors, including soil texture [9], planting date [10], temperature [11], and soil pH [12]. These factors, combined with significant genotype–environment interactions [13,14,15], create variable responses that cannot be addressed solely through conventional seed rotation approaches.

Northeastern Thailand offers substantial untapped potential for soybean cultivation expansion through strategic integration with existing agricultural systems. The region’s dual cropping calendar creates complementary production windows that enable innovative soybean-rice and soybean–sugarcane rotation systems. In lowland areas, rice cultivation follows a distinct seasonal pattern, with harvest completed by November–December, leaving fields fallow until the next planting season, May–June. This 5–6 month period (December to April) presents an ideal window for dry-season soybean production. Research demonstrates that rice grown after legume crops without nitrogen fertilizer produced 13–23% higher grain yield than rice after fallow rotation [16].

Upland areas following sugarcane harvest present equally valuable opportunities. Sugarcane harvest occurs primarily from December to April, after which fields remain fallow until replanting in the following rainy season (June–October). This extended 4–6 months period coincides with the rainy season, providing natural irrigation for soybean cultivation. Studies have shown that soybean varieties can produce 9 t/ha above-ground dry weight, containing 301 kg N/ha which contributed to soil N stores, with potential reductions in fertilizer application rates up to approximately 100% in the first ratoon, and 60%, 25% and 10% in subsequent ratoons [17]. The complementary nature of these two systems enables an innovative integrated seed rotation strategy that requires varieties with stable performance across both environments. This bi-directional seed flow addresses seasonal seed shortage constraints while maintaining genetic purity but demands varieties with demonstrated stability across diverse environmental conditions to ensure system reliability.

Improving soybean yield through seed rotation presents multiple challenges, primarily due to significant genotype-by-environment interactions [8]. Environmental conditions vary significantly across Northeastern Thailand, with soil textures ranging from sandy loam to clay, pH values between 5.5 and 7.2, and considerable variation in nutrient levels [8]. Varieties that exhibit extensive genotype-by-environment interactions may perform exceptionally in specific environments but fail to maintain consistent yields across the diverse conditions encountered in seed rotation systems. Modern stability analysis relies on advanced statistical approaches, particularly Additive Main Effects and Multiplicative Interaction (AMMI) and Genotype plus genotype-by-environment interaction (GGE) biplot models. AMMI analysis decomposes the genotype-by-environment interaction into principal components through Principal Component Analysis (PCA). GGE biplot analysis offers complementary advantages by incorporating the “which-won-where” pattern, environment ranking, and mean versus stability relationships [18,19]. Research demonstrates that genotypes closer to the center of biplot models tend to exhibit greater stability [20]. Several studies have used AMMI and GGE-biplot analyses to aid breeders in selecting the best genotypes for specific or general adaptability to different environments before commercial release under multi-environmental trials [21,22,23,24,25].

The soybean variety Morkhor 60 (previously designated as 35*sj-32) has demonstrated promising yield potential exceeding commercial varieties in preliminary evaluations across rainy-season uplands and dry-season lowlands in Northeastern Thailand [8]. This variety represents a potential breakthrough for implementing the innovative integrated rotation strategy, capitalizing on fallow periods in both production systems. While Morkhor 60 shows promising yield potential, a comprehensive evaluation of its yield stability across these contrasting post-sugarcane and post-rice environments remains incomplete. Successful implementation of this bi-directional system requires varieties with demonstrated consistent performance across diverse environmental conditions.

Therefore, this study aimed to evaluate the yield performance, stability, and quality attributes of Morkhor 60 compared to commercial soybean varieties (SJ 5, 223*Lh-85, and CM 60) across thirteen environmentally diverse locations in Northeastern Thailand, with emphasis on identifying its suitability for seed rotation systems requiring consistent performance across contrasting environments. It was hypothesized that this comprehensive evaluation would provide evidence for its suitability in regional seed rotation systems and offer a viable strategy to reduce national soybean seed shortages while enhancing domestic production.

## 2. Results

### 2.1. Environmental Variation

The thirteen environments exhibited substantial variation in soil properties and growing conditions, forming distinct environmental groups (Table 1). Sandy loam soils with pH 5.5–6.5 predominated in Khon Kaen Province and Phu Khiao District (KK-R-22, PK-R-22, KK-D-22, KK-R-23, PK-R-23, KK-D-23, PK-D-23), while clay soils with pH ≥ 7.0 characterized Chum Phae District environments (CP-R-22, CP-D-22-1, CP-D-22-2, CP-R-23, KS-R-23, CP-D-23). Available phosphorus ranged from 2.08 to 96.00 mg/kg, and exchangeable potassium varied from 25.03 to 830.03 mg/kg, reflecting the diverse soil fertility conditions across the region. (Table 1). These contrasting environments caused variations in grain yield, in which KK-D-22, PK-R-23, KK-D-23, and PK-R-22 produced the highest grain yields (Table 1).

### 2.2. Sources of Variance for Yield Components and Grain Yield of Four Soybean Genotypes Across 13 Environments

The combined analysis of variance across thirteen environments revealed highly significant effects (*p* < 0.01) for environment (E), genotype (G), and genotype-by-environment interactions (G × E) for all measured traits (Table 2). Environmental factors exhibited the most pronounced influence on soybean performance, with highly significant effects on all traits, including grain yield, yield components, and vegetative characteristics (Table 2). This substantial environmental variation confirms the diverse testing conditions essential for comprehensive stability evaluation (Table 1). Significant genotypic differences (*p* < 0.01) were observed for all traits, with particularly notable effects for grain yield, pods per branch, and pods per plant, indicating strong genetic control over these agronomic traits (Table 2). The highly significant G × E interactions (*p* < 0.01) for all traits (Table 2) demonstrate that varieties responded differently to diverse environmental conditions, providing strong justification for detailed stability analysis to identify varieties suitable for integrated seed rotation systems. Experimental precision was confirmed by acceptable coefficient of variation (CV) values ranging from 3.64% to 22.62%, with grain yield showing a CV of 7.97%, confirming reliable data collection across diverse conditions. The significant G × E interactions provide strong justification for conducting detailed stability analysis using AMMI and GGE biplot analyses to identify varieties suitable for integrated seed rotation systems.

### 2.3. Yield Performance of Four Soybean Genotypes Across 13 Environments

The comprehensive yield evaluation across thirteen environmentally diverse locations during 2022–2023 provided evidence of Morkhor 60’s competitive yield performance and consistent promise over commercial varieties (Table 3 and Table 4). This multi-environment assessment encompassed rainy season (post-sugarcane upland) and dry season (post-rice lowland) production systems.

The 2022 experimental results demonstrated highly significant yield differences (*p* < 0.01) among varieties across six environments (Table 3). During rainy season trials (KK-R-22, CP-R-22, PK-R-22), Morkhor 60 achieved favorable yields, with the highest recorded yield of 1954 kg/ha in environment PK-R-22. In KK-R-22, Morkhor 60 produced 1428 kg/ha, while CP-R-22 yielded 1213 kg/ha. The rainy season average for Morkhor 60 was 1532 kg/ha, demonstrating consistent performance across diverse upland conditions. Dry season evaluation (KK-D-22, CP-D-22-1, CP-D-22-2) revealed Morkhor 60’s competitive productivity under irrigated systems. Environment KK-D-22 produced the highest dry season yield of 2046 kg/ha for Morkhor 60, followed by CP-D-22-1 with 1789 kg/ha, and CP-D-22-2 with 1302 kg/ha. The dry season average reached 1712 kg/ha, indicating promising adaptation to controlled irrigation conditions.

Overall, the 2022 performance analysis revealed Morkhor 60’s favorable yield, promising, achieving the highest mean yield of 1622 kg/ha across all environments. This represented significant yield advantages of 2.6% over SJ 5 (1581 kg/ha), 12.8% over CM 60 (1438 kg/ha), and 27.1% over 223*Lh-85 (1276 kg/ha) (Table 3).

The 2023 trials across seven environments further substantiated Morkhor 60’s exceptional yield capability. Rainy season evaluation (KK-R-23, CP-R-23, PK-R-23, KS-R-23) culminated in Morkhor 60 achieving the highest individual yield recorded during the entire study: 2330 kg/ha in environment PK-R-23 (Table 4). Additional rainy season yields included 1434 kg/ha in KK-R-23, 1101 kg/ha in CP-R-23, and 818 kg/ha in KS-R-23, averaging 1421 kg/ha across rainy season environments. Dry season trials (KK-D-23, CP-D-23, PK-D-23) demonstrated Morkhor 60’s continued yield excellence. Environment KK-D-23 produced 1733 kg/ha, CP-D-23 yielded 1294 kg/ha, and PK-D-23 generated 979 kg/ha, with a dry season average of 1335 kg/ha. Despite more challenging conditions in 2023, Morkhor 60 maintained high yield across all environments except KK-D-23 and PK-D-23.

The 2023 comprehensive evaluation confirmed Morkhor 60’s sustained yield is promising, achieving the highest mean yield of 1384 kg/ha across seven environments. This performance represented yield advantages of 5.1% over SJ 5 (1317 kg/ha), 7.5% over CM 60 (1287 kg/ha), and 17.4% over 223*Lh-85 (1179 kg/ha).

The two-year yield analysis encompassing 13 diverse environments demonstrated Morkhor 60’s exceptional yield consistency and superiority. Morkhor 60 maintained the highest average yields in both experimental years: 1622 kg/ha in 2022 and 1384 kg/ha in 2023. Peak individual yields reached 2330 kg/ha during rainy season cultivation and 2046 kg/ha under dry season irrigation, representing the highest yields achieved by any variety in their respective seasons (Table 3 and Table 4). This consistent high-yield performance across diverse environmental conditions established Morkhor 60’s promise for integrated seed rotation systems requiring reliable productivity across contrasting post-sugarcane and post-rice cultivation scenarios.

### 2.4. Yield Stability of Four Soybean Genotypes

**Figure 1:**
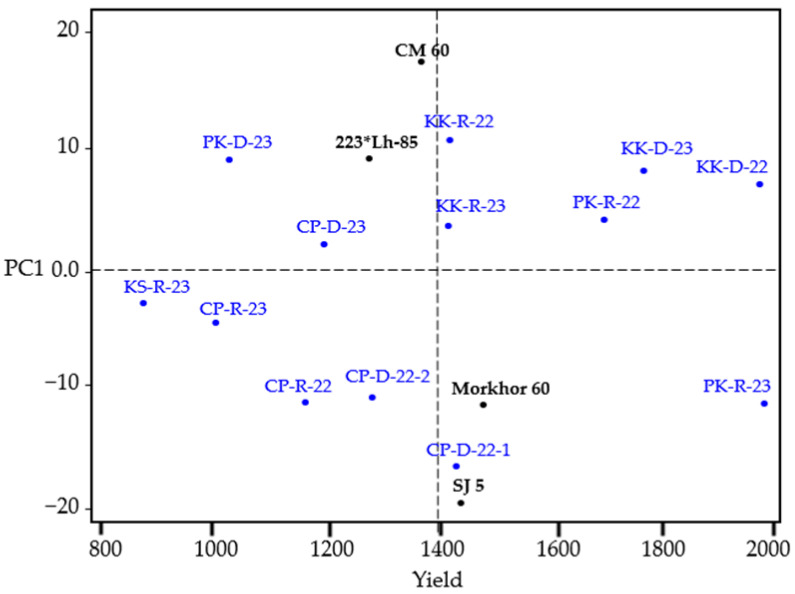
AMMI1 biplot analysis demonstrating yield performance and stability of four soybean genotypes across thirteen diverse environments in Northeastern Thailand. Morkhor 60 (positioned furthest right) shows the highest average yield and superior stability (closest to PC1 axis), confirming its broad adaptability for integrated seed rotation systems. Genotypes are shown in black, and environments are shown in blue text.

The AMMI1 biplot demonstrated a positive relationship between PC1 and yield. The vertical axis represents the average yield across all environments. Environments located to the right of the yield axis have higher average yields, namely, environments KK-R-22, KK-R-23, CP-D-22-1, PK-R-22, KK-D-23, KK-D-22, and PK-R-23, with the rightmost environment having the highest average yield. Similarly, the genotypes Morkhor 60 and SJ 5 are positioned to the right of the yield axis, indicating higher average yields than the overall genotype average. However, Morkhor 60 is placed farthest to the right, indicating the highest average yield. Additionally, Morkhor 60 is closest to the PC1 axis, suggesting that it has lower genotype-by-environment interaction (G × E) than SJ 5. This indicates that Morkhor 60 has the highest average yield and is less responsive to environmental variations (Figure 1).

**Figure 2:**
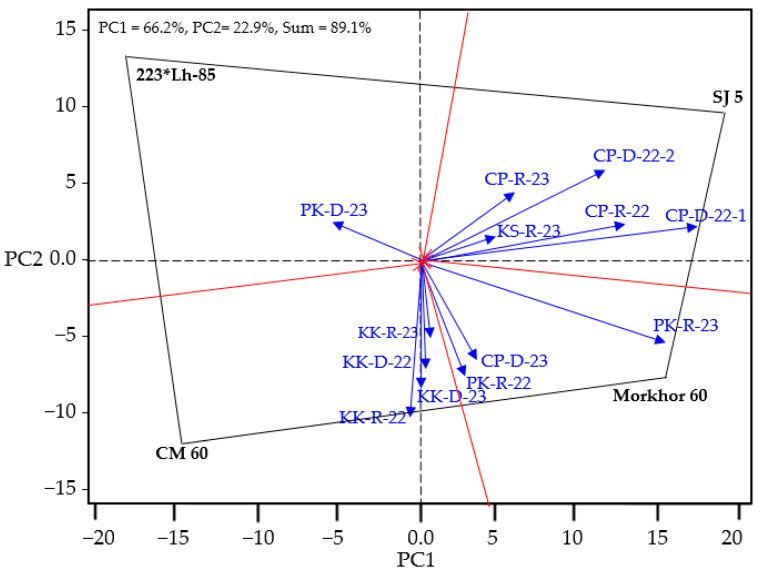
GGE biplot analysis identifying mega-environments and genotype adaptability patterns across thirteen test locations. The first two principal components explain 89.1% of genotype-by-environment variance, with Morkhor 60 showing broad adaptability across multiple environmental sectors, positioning it as ideal for integrated rotation systems between post-sugarcane and post-rice areas. Genotypes are shown in black, and environments are shown in blue text.

The first two principal components (PC1 and PC2) in the GGE-blot analysis could explain 89.1% of the variance in G × E. There were four main environmental groups. Environment group 1 consisted of genotype SJ 5, the apecis of environments CP-R-22, CP-D-22-1, CP-D-22-2, CP-R-23, and KS-R-23 as the first large environment. Environment group 2 consisted of genotype Morkhor 60, the apecis of environment group 2, and PK-R-22, PK-R-23, and CP-D-23. Environment group 3 consisted of genotype CM 60 and the apecis of environments KK-R-22, KK-D-22, KK-R-23, and KK-D-23. Environment group 4, which consisted of genotype 223*Lh-85, was the apex of environment PK-D-23 and was not considered the main environment. Genotype Morkhor 60 was positioned at the apex of an environment group that was intermediate between those dominated by SJ 5 and CM 60. This suggests that Morkhor 60 is a broadly adaptable genotype capable of thriving in multiple environments. Furthermore, most environments formed approximately 90-degree angles with both Morkhor 60 and SJ 5 in the GGE biplot, indicating that both genotypes exhibit relatively high yields across different environments. However, Morkhor 60 showed slightly better performance in environments PK-R-22, PK-R-23, and CP-D-23, while SJ 5 performed better in environments CP-R-22, CP-D-22-1, and CP-D-22-2 (Figure 2).

### 2.5. Variation in Chemical Components in Four Soybean Genotypes

The combined analysis of variance across seven environments revealed highly significant effects (*p* < 0.01) for environment (E), genotype (G), and genotype-by-environment interactions (G × E) for all measured traits (Table 5). Environmental factors exhibited the most pronounced influence on soybean chemical components, with highly significant effects on all traits, including protein, oil, fiber, and ash percentage (Table 5).

The results of the chemical analysis revealed statistically significant differences in the chemical composition of soybeans, specifically in ash, crude fiber, fat, and protein, due to ecological and genotype influences (Table 6). Additionally, an interaction between genotype and environment was observed. Among the soybean genotypes, SJ 5 had the highest average protein content at 40.74%, followed by Morkhor 60 (39.63%), CM 60 (38.20%), and 223*Lh-85 (35.53%), respectively. However, regarding fat composition, CM 60 had the highest average fat content at 16.65%, while 223*Lh-85, Morkhor 60, and SJ 5 had 15.72%, 14.66%, and 13.38%, respectively. These results indicate an inverse relationship between protein and fat content in soybean cultivars and also demonstrate the quality characteristics of the ‘Morkhor 60’ variety. Regarding fiber content, Morkhor-60 (14.70%) and SJ 5 (15.29%) showed higher values compared to CM 60 (13.54%) and 223*Lh-85 (13.84%). Higher fiber content is generally favorable for animal feed applications, improving digestibility and nutritional value for livestock. However, for human consumption and food processing, moderate fiber levels are preferred to maintain palatability and processing characteristics.

Ash content represents the mineral content of soybeans, with higher values indicating greater mineral density. CM 60 exhibited the highest ash content (6.31%), followed by 223*Lh-85 (5.66%), SJ 5 (5.63%), and Morkhor 60 (5.53%). While Morkhor 60 showed the lowest ash content, all varieties fell within acceptable ranges (5.0–6.5%) for both food and feed applications. The differences in ash content may reflect varietal differences in mineral accumulation capacity and environmental adaptation strategies.

## 3. Discussion

Soybean production across diverse environments in Northeastern Thailand faces significant challenges due to substantial environmental variability, which critically affects yield performance and stability [8]. The comprehensive analysis of Morkhor 60 across thirteen environmentally diverse locations during 2022–2023 provides crucial insights into the potential for implementing effective seed rotation systems in post-sugarcane and post-rice production areas. The environmental data collected across these test locations revealed considerable variation in soil texture, pH values, nutrient levels, and planting dates (Table 2), resulting in significant differences in yield and yield components among genotypes and environments (Table 3).

### 3.1. Environmental Effects and Genotype Performance in Soybean Production

The substantial environmental influence observed in this study aligns with research by Amogne et al. [26], who reported that environmental conditions account for most yield variation in multi-environment soybean trials. Similarly, Sritongtae et al. [8] documented that ecological influences on soybean yield in Northeastern Thailand reached 97.68%, while genotypic effects contributed only 1.40%, underscoring the critical importance of environmental adaptation in this region.

Analysis of the environmental conditions reveals that soil texture, pH, and seasonal water management are the primary determinants of soybean productivity in Northeastern Thailand. Sandy loam soils with pH values between 5.5 and 6.5 provide the optimal combination for soybean cultivation due to their superior drainage characteristics and balanced nutrient availability. These soils facilitate efficient nutrient uptake and utilization when supplemented with balanced NPK fertilization, as demonstrated by Bashir et al. [9]. The pronounced difference in productivity between sandy loam and clay soil environments reflects the critical importance of soil physical properties in determining crop performance potential.

Significant agronomic differences between soil types directly influence the biological processes critical for soybean productivity. Clay soils create suboptimal growing conditions through poor drainage, restricted root development, and reduced soil aeration, which severely compromise soybean nodulation and nitrogen fixation efficiency. The symbiotic relationship between soybeans and *B. japonicum* bacteria requires adequate oxygen availability for optimal function [27], making soil aeration a critical limiting factor in heavy clay soils. Soil pH represents another crucial biological determinant affecting plant–microbe interactions and nutrient availability. Alkaline conditions (pH > 7.0) create multiple physiological constraints, including reduced nutrient solubility, impaired rhizobial effectiveness, and compromised root function [28]. This observation aligns with findings by Uguru et al. [12], who reported that optimal soybean growth occurs in slightly acidic to neutral pH ranges (6.0–7.0), where both nutrient availability and biological nitrogen fixation are maximized.

Distinct agronomic and biological differences characterize the two production seasons, creating contrasting opportunities and limitations. Rainy season production operates under natural precipitation patterns, resulting in higher yield variability due to irregular rainfall distribution and waterlogging risks in clay soils. The biological advantage of rainy season cultivation lies in enhanced soil microbial activity and natural moisture availability, promoting vigorous vegetative growth and nodulation [5]. However, this system faces challenges from unpredictable weather patterns and increased disease pressure under humid conditions. Dry season production benefits from controlled irrigation management, leading to more predictable and consistent growing conditions. The biological processes operate under managed water stress, promoting more efficient water use and compact plant architecture. Temperature regimes during dry season cultivation are often more favorable for reproductive development, with cooler temperatures during pod filling enhancing seed quality [6]. However, dry season production faces constraints from limited water availability, suboptimal planting dates determined by primary crop harvest schedules, and reduced soil biological activity under drier conditions. The interaction between seasonal conditions and soil types creates distinct micro-environments that differentially affect plant physiology [9]. Sandy loam soils perform consistently well across both seasons due to their balanced water-holding capacity and drainage characteristics, while clay soils show greater seasonal variation due to waterlogging during rainy seasons and hardpan formation during dry periods [27].

The significant genotypic differences observed across all measured traits demonstrate the importance of variety selection in maximizing productivity under diverse environmental conditions [13,14,15]. Morkhor 60’s consistent performance across contrasting environments indicates robust genetic mechanisms for environmental adaptation, including enhanced stress tolerance and stable physiological processes under varying conditions [8,20]. The stability analysis using AMMI1 and GGE biplot methodologies revealed critical insights into genotype–environment interactions and adaptive capacity. The AMMI1 model demonstrated that Morkhor 60 exhibits minimal genotype-by-environment interaction, indicating promising phenotypic stability across diverse growing conditions [29]. This stability reflects underlying genetic mechanisms that maintain consistent performance despite environmental fluctuations, a crucial characteristic for successful seed rotation systems. The GGE biplot analysis provided complementary insights into mega-environment classification and genotype adaptability patterns. The explanation of 89.1% of genotype-by-environment interaction variance by the first two principal components indicates robust model fit and reliable interpretation of genotype performance patterns [30]. Morkhor 60’s intermediate positioning between environmental sectors dominated by other varieties demonstrates broad adaptability and balanced performance across multiple environmental conditions [31].

The central positioning of Morkhor 60 in the GGE biplot indicates exceptional stability characteristics, as varieties closer to the biplot center exhibit greater stability across diverse environments [32]. This stability, combined with superior average performance, represents the ideal combination for integrated seed rotation systems requiring consistent productivity across contrasting post-sugarcane and post-rice cultivation environments. The stability analysis confirms that Morkhor 60 possesses the genetic architecture necessary for successful implementation in bi-directional seed rotation systems. Unlike varieties that show strong adaptation to specific environments but poor performance in others, Morkhor 60’s broad adaptability ensures reliable seed production across both upland rainy season and lowland dry season production windows, addressing the critical seed shortage constraint while maintaining genetic quality and vigor.

### 3.2. Soybean–Rice Cropping System Integration

Integrating Morkhor 60 into rice-based cropping systems represents a significant opportunity for maximizing land utilization efficiency during fallow periods. Rice cultivation in Northeastern Thailand follows a distinct seasonal pattern, with harvest typically completed by November–December, leaving lowland fields fallow until the next planting season in May–June. This 5–6 month follow-up period coincides perfectly with the dry season growing window (December–April), where Morkhor 60 achieved substantial yields ranging from 818 to 2046 kg/ha across dry season environments.

The benefits of legume–rice rotation systems extend beyond immediate yield considerations to include significant improvements in soil fertility and subsequent rice productivity. Research by Rahman et al. [16] demonstrated that rice grown after legume crops without nitrogen fertilizer produced 13–23% higher grain yield than rice after fallow, attributed to residual nitrogen from biological nitrogen fixation. The nitrogen fixation capacity of soybeans, estimated at 30–40 kg N per ton of shoot dry matter [5], provides substantial nitrogen input for subsequent rice crops, potentially reducing fertilizer requirements by 40–60 kg N/ha [33]. The residual effects of soybean cultivation on subsequent rice crops are well-documented, with studies showing that decomposition of soybean residues contributes significant amounts of organic matter and nitrogen to the soil system, with N contributions ranging from 40 to 80 kg N/ha, depending on biomass production and environmental conditions [34]. This residual effect can persist for multiple cropping seasons, providing sustained benefits to the rice–soybean rotation system [35].

### 3.3. Soybean-Sugarcane Cropping System Potential

Integrating Morkhor 60 into sugarcane-based systems offers equally promising opportunities for enhancing agricultural sustainability and productivity. Sugarcane harvest in Northeastern Thailand occurs primarily from December to April, after which fields remain fallow until replanting in the following late rainy season (October–November). This extended follow-up period of 4–6 months coincides with the rainy season growing window, during which Morkhor 60 demonstrated exceptional performance, achieving peak yields of 2330 kg/ha in environment PK-R-23 (Table 4).

Research by Park et al. [17] demonstrated that soybean cultivation in sugarcane systems can contribute substantial amounts of nitrogen to soil reserves, with varieties producing up to 301 kg N/ha in above-ground biomass. This nitrogen contribution can significantly reduce fertilizer requirements for subsequent sugarcane ratoons, with potential reductions of up to 100% in the first ratoon and 60%, 25%, and 10% in subsequent ratoons, representing substantial cost savings for farmers. The environmental benefits extend beyond nitrogen fixation, including improved soil health, enhanced microbial diversity, and reduced pest and disease pressure through crop rotation effects [36]. Additionally, the organic matter contribution from soybean residues improves soil water-holding capacity and nutrient retention, which is critical for sustainable sugarcane production [37]. The breakdown of soybean root nodules also releases significant amounts of fixed nitrogen into the soil profile, with estimates ranging from 25 to 50% of total fixed nitrogen becoming available to subsequent crops [38].

### 3.4. Seed Rotation System Implementation

**Figure 3:**
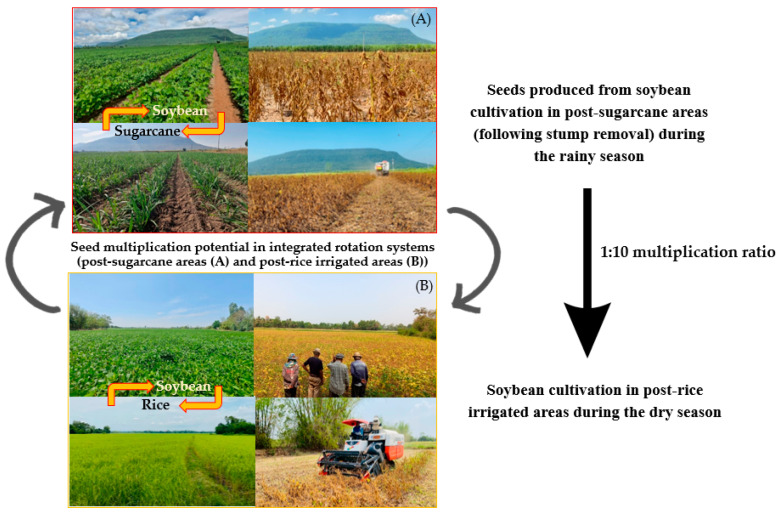
Integrated bi-directional seed rotation system utilizing soybean variety ‘Morkhor 60’ between post-sugarcane upland environments and post-rice lowland environments in Northeastern Thailand, demonstrating the complementary seasonal production windows and seed flow patterns that address regional seed shortage constraints.

The promising yield stability demonstrated by Morkhor 60 across both post-sugarcane upland and post-rice lowland environments positions it as an ideal candidate for innovative seed rotation systems in Northeastern Thailand. The bi-directional seed flow enabled by this variety’s broad adaptability creates a self-sustaining system where rainy season production in upland areas supplies seeds for dry season cultivation in lowland areas, and vice versa (Figure 3). The seed rotation concept becomes particularly valuable when considering the storage limitations of soybean seeds, which typically maintain high germination rates for only 12–18 months under tropical conditions [7].

The Morkhor 60-based rotation system ensures fresh seed availability while maintaining genetic purity and vigor by enabling two yearly production cycles in complementary environments. The system’s effectiveness is enhanced by the variety’s consistent performance across diverse soil types and seasonal conditions, reducing the risk of crop failure and ensuring reliable seed production [39]. Seeds with high vigor and germination rates are essential for successful establishment, particularly under variable environmental conditions [40]. Based on our environmental analysis, priority target areas for soybean expansion include post-sugarcane sandy loam upland environments in Phu Khiao district, Chaiyaphum province, during the rainy season, and lowland paddy fields with adequate irrigation in Chum Phae district, Khon Kaen province, during the dry season (Figure 3). The implementation of soybean-based rotation systems offers significant advantages for enhancing agricultural productivity and maintaining long-term soil health in these target environments [41].

### 3.5. Quality Characteristics and Environmental Benefits

The chemical composition analysis revealed essential quality characteristics of Morkhor 60, with protein content of 39.63% and oil content of 14.66%, representing a favorable balance for food and feed applications. The inverse relationship between protein and oil content observed across all genotypes is consistent with established soybean biochemistry [42,43]. The stability of yield and quality characteristics across diverse environments enhances the market potential of Morkhor 60 for consistent supply chain development [44].

Implementing Morkhor 60-based seed rotation systems offers significant environmental benefits beyond immediate production gains. The nitrogen fixation capacity of soybeans reduces the need for synthetic nitrogen fertilizers, decreasing greenhouse gas emissions associated with fertilizer production and application. Studies have shown that legume-based rotations can reduce N_2_O emissions by 35% compared to continuous cereal cropping systems [6,45]. The diversification provided by soybean integration enhances biodiversity above and below ground, supporting beneficial soil microorganisms and natural pest control mechanisms [46]. This biological diversity strengthens system resilience against environmental stresses and reduces dependence on external inputs [47]. Economic benefits include reduced input costs, improved soil productivity, and diversified farmer income streams, contributing to enhanced rural livelihoods and food security [48].

### 3.6. Yield Levels and Economic Viability

The observed yields (749–2330 kg/ha) represent realistic production levels for Northeastern Thailand’s environmental conditions and existing farming practices, rather than research station optimal conditions. Several factors contribute to these yield levels: (1) Rainfed production limitations during wet season with irregular precipitation patterns, (2) Marginal soil fertility in post-harvest rotation systems, (3) Limited fertilizer inputs following traditional low-input farming practices, and (4) Suboptimal planting dates constrained by primary crop (rice/sugarcane) harvest schedules [8].

These yields remain economically viable for smallholder farmers due to the following factors: (1) Minimal production costs in rotation systems utilizing residual soil fertility, (2) Premium prices for locally-produced soybeans (15–20% above import prices), (3) Additional income during traditional fallow periods, and (4) Nitrogen fixation benefits reducing fertilizer costs for subsequent crops [5,49,50]. Economic analysis indicates positive net returns of THB 8000–15,000/ha (approximately USD 230–430/ha) even at observed yield levels, making soybean cultivation profitable within existing farming systems.

### 3.7. Future Research Directions

Future research priorities should focus on optimizing management practices for Morkhor 60 cultivation [51] and examining the long-term effects of these rotation systems on soil health and productivity [52]. Additional research directions include molecular characterization of the stability mechanisms and evaluation of the variety’s performance in other tropical regions with similar environmental conditions.

## 4. Materials and Methods

### 4.1. Plant Material and Experiment Design

Four soybean genotypes were evaluated in this study: two varieties from the Department of Agriculture (SJ 5 and CM 60), one certified variety, Morkhor 60 (previously breeding line 35*sj-32), and one breeding line (223*Lh-85) derived from several crosses through pedigree selection since 2004 by The Plant Breeding Research Center for Sustainable Agriculture, Khon Kaen University [8]. All genotypes belong to intermediate maturity (90–110 days) and are suitable for planting in a rotation cropping system previously reported by Sritongtae et al. [8]. The initial seed of each genotype was provided by the Plant Breeding Research Center for Sustainable Agriculture, Khon Kaen University.

**Figure 4:**
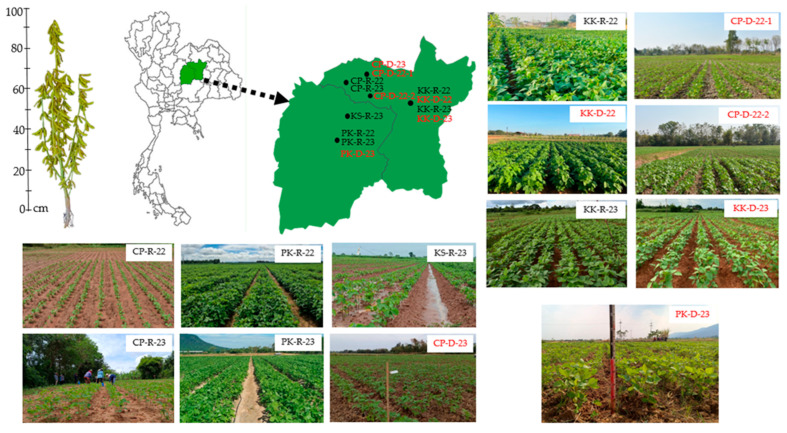
Map of thirteen experimental sites across Northeastern Thailand showing Morkhor 60 soybean performance at the R6 growth stage. Site names for rainy season trials are in black text, and dry season trials are in red.

Experiments were conducted across thirteen environments in two provinces and four districts of Northeastern Thailand during 2022–2023. Environment selection was based on representativeness of major soil types and cropping systems in the region, ensuring comprehensive evaluation across post-sugarcane upland and post-rice lowland production areas (Figure 4, Table 1). Environmental conditions varied significantly by location, planting time, and soil characteristics, providing robust testing conditions for stability analysis. The Thai Meteorological Department [53] defines Thailand’s dry season as typically lasting from November to April, beginning with the end of the rainy season. The rainy season occurs from mid-May to mid-October. This seasonal pattern is particularly evident in Northeastern Thailand, which Goto et al. [54] and Polthanee and Srisutham [55] describe as having a semi-humid tropical climate characterized by distinct rainy (May–October) and dry (November–April) seasons.

The location code were assigned as (KK-R-22) KKU, Khon Kaen Province, during rainy season of 2022 (16°28′23.4″ N, 102°48′30.0″ E); (CP-R-22) Chum Phae District, Khon Kaen Province, during rainy season 2022 (16°38′28.5″ N, 101°57′54.9″ E); (PK-R-22) Phu Khiao District, Chaiyaphum Province, during rainy season 2022 (16°18′24.1″ N, 102°11′51.2″ E); (KK-D-22) KKU, Khon Kaen Province, during dry season 2022 (16°28′20.5″ N, 102°48′37.3″ E); (CP-D-22) Chum Phae District, Khon Kaen Province, during dry season 2022 (16°38′09.6″ N, 101°57′59.3″ E); (CP-D-22) Chum Phae District, Khon Kaen Province, during dry season 2022 (16°38′31.6″ N, 101°57′57.5″ E); (KK-R-23) KKU, Khon Kaen Province, during rainy season 2023 (16°28′23.4″ N, 102°48′30.0″ E); (CP-R-23) Chum Phae District, Khon Kaen Province, during rainy season 2023 (16°38′22.2″ N, 101°57′33.1″ E); (PK-R-23) Phu Khiao District, Chaiyaphum Province, during rainy season 2023 (16°18′16.9″ N, 102°11′20.9″ E); (KS-R-23) Khok Sa-at District, Chaiyaphum Province, during rainy season 2023 (16°27′00.5″ N, 102°05′56.1″ E); (KK-D-23) KKU, Khon Kaen Province, during dry season 2023 (16°28′20.5″ N, 102°48′37.3″ E); (CP-D-23) Chum Phae District, Khon Kaen Province, during dry season 2023 (16°27′35.6″ N, 102°17′48.4″ E); (PK-D-23) Phu Khiao District, Chaiyaphum Province, durings dry season 2023 (16°18′15.0″ N, 102°11′06.4″ E) (Figure 4, Table 1).

A randomized complete block design with four replications was employed at each location. At most locations, experimental plots measured 54 m^2^ (6 × 9 m), with plant spacing of 25 cm within rows and 50 cm between rows. However, at three locations (E3, E9, and E10), the experimental plots differed in size to match the existing sugarcane bed configuration. At these locations, experimental plots consisted of approximately three beds, measuring 55.5 m^2^ (5.55 × 10 m), with the same plant spacing of 25 cm within rows and 50 cm between rows. Planting was strategically conducted during two distinct seasons: the rainy season (July–August) for post-sugarcane upland environments and the dry season (December–April) for post-rice lowland environments.

Standardized crop management protocols were implemented across all locations to minimize management-related variation. Seeds with germination rates of 80–90% were used for initial planting at 5 seeds per hole. At 15 days after planting (DAP), plants were thinned to maintain three plants per hole to ensure uniform plant density. Weed control was performed manually to minimize herbicide interference with crop development. A standardized fertilizer management program was implemented across all locations. Initial fertilizer application provided 23.44 kg/ha each of N, P_2_O_5_, and K_2_O at planting. A second application at 30 DAP supplied an additional 23.44 kg/ha of each nutrient, resulting in a total application rate of 46.88 kg/ha. Recommended herbicides and insecticides were applied based on local pest and weed pressure. Supplemental irrigation was provided during the dry season to ensure adequate soil moisture for crop development using sprinkler systems at upland stations and furrow irrigation applied 4–5 times during the crop cycle in lowland paddy fields.

### 4.2. Data Collection

Comprehensive soil characterization was conducted at each location before planting. Soil samples were collected at 0–15 cm depth from four random locations within each experimental plot for chemical analysis. Standard analytical methods were employed. Soil texture was determined using the hydrometer method [56]. Soil pH was measured using a 1:1 ratio of soil to H_2_O [57], soil total nitrogen (N) content was determined by micro Kjeldahl digestion [58], and available phosphorus (*p*) was measured following Bray II extraction [59]. At the same time, exchangeable potassium (K) was analyzed using a flame photometer after 1 M ammonium acetate extraction at pH 7 [60].

Grain yield determination followed standardized protocols using randomly selected 1 m^2^ quadrats within each plot, with four sampling points per plot containing 15 holes with three plants each. Harvesting occurred during October–November for the rainy season and March–April for the dry season, depending on crop maturity. Grain yields from individual plots were adjusted to 13% grain moisture content and converted to kg per hectare.

Plant morphological parameters, including the number of nodes, branches, and pods, were counted from 10 randomly selected plants per plot and calculated on a per-plant basis. Additional measurements recorded from the same plants included the number of pods on the main stem and the number of pods on branches. The number of grains per pod was also recorded from 10 randomly selected pods per plant in each plot. One hundred-grain weight was also determined using viable grains from each replication [8].

Nutritional analysis followed modified standards at the Animal Nutrition Unit, Department of Animal Science, Khon Kaen University, Khon Kaen, Thailand.

Crude fat (ether extract, EE), crude fiber (CF), and ash content were determined using the Weende method according to AOAC standards [61] with slight modifications as described below.

Determination of EE: A 5.0 g powdered sample was extracted in 100 mL diethyl ether and agitated for 24 h using an orbital shaker. The filtrate was collected in the same flask, equilibrated with an additional 100 mL of diethyl ether, and shaken for another 24 h. The ether was concentrated to dryness using a steam bath, then dried in an oven at 60 °C for 30 min. The ether extract weight was determined and calculated as a percentage of the sample weight using Formula (1) [62]:

(1) $$\mathrm{Crude}\;\mathrm{fat}\;(\%)\;=\;\frac{\mathrm{Weight}\;\mathrm{of}\;\mathrm{flask}\;\mathrm{with}\;\mathrm{fat}\;-\;\mathrm{Weight}\;\mathrm{of}\;\mathrm{empty}\;\mathrm{flask}}{\mathrm{Weight}\;\mathrm{of}\;\mathrm{sample}}\;\times 100\;$$

Determination of CF: A 5.0 g powdered sample was treated with 100 mL of 1.25% H_2_SO_4_ for 30 min and filtered under pressure. The residue was washed with hot water and then treated with 100 mL of 1.25% NaOH solution, following the same procedure. The remaining residue was dried at 100 °C and incinerated in a muffle furnace at 550 °C for 5 h. The fiber weight was determined and calculated as a percentage of the sample weight using Formula (2) [63]:

(2) $$\mathrm{Crude}\;\mathrm{fiber}\;(\%)\;=\;\frac{\left(\mathrm{Weight}\;\mathrm{of}\;\mathrm{crucible}\;+\;\mathrm{Ash}\right)-\mathrm{Weight}\;\mathrm{of}\;\mathrm{crucible}\;+\;\mathrm{sample}\;\mathrm{after}\;\mathrm{washing},\mathrm{boiling}\;\mathrm{and}\;\mathrm{drying}}{\mathrm{Weight}\;\mathrm{of}\;\mathrm{sample}}\;\times \;100\;$$

Determination of ash: A porcelain crucible was dried at 105 °C for 1 h, and then, a 5.0 g powdered sample was placed in the crucible. The sample was initially ashed at 250 °C for one hour, followed by ashing at 550 °C for five hours in a muffle furnace. After cooling in a desiccator, the ash weight was determined and calculated as a percentage of the sample weight using Formula (3) [63]:

(3) $$\mathrm{Ash}\;(\%)\;=\frac{(\mathrm{Weight}\;\mathrm{of}\;\mathrm{crucible}\;+\;\mathrm{Ash})\;-\;\mathrm{Weight}(g)\mathrm{of}\;\mathrm{empty}\;\mathrm{crucible}}{\mathrm{Weight}\;\mathrm{of}\;\mathrm{sample}}\times \;100\;$$

Total nitrogen was determined using the Kjeldahl method according to AOAC standards [61]. Approximately 10 g of potassium sulfate and 0.5 g of copper sulfate were added to a 25 mL concentrated sulfuric acid flask. The flask was heated gently in a digestion chamber until the contents became clear, then cooled and diluted with 200 mL of distilled water. The solution was distilled until all ammonia was collected in standard sulfuric acid, which was then back-titrated with standard NaOH to determine the amount of acid used to neutralize the ammonia from the digested material. A blank sample was processed simultaneously. Total nitrogen was calculated using Formula (4) [61], and crude protein (CP) was subsequently calculated from the total nitrogen using Formula (5) [61].

(4) $$\mathrm{Total}\;\mathrm{nitrogen}\;(\%)=\frac{1.4(B-A)N}{\mathrm{Weight}\;\mathrm{of}\;\mathrm{sample}}$$

(5) Crude protein (%) = total nitrogen × 6.25

where B = volume of N/10 NaOH for blank; A = volume of N/10 NaOH used for sample, and N = normality of standard NaOH

### 4.3. Statistical Analysis

Yield data were analyzed using STATISTIX 10 software (Copyright© 1985–2013, Analytical Software 2105 Miller Landing Rd. Tallahassee, FL, USA) through a combined analysis of variance (ANOVA) for a randomized complete block design across environments. The four genotypes were evaluated across all thirteen environments, with treatment means compared using least significant difference (LSD) at *p* = 0.05. Yield stability was assessed using two complementary analytical approaches. (1) Additive main effects and multiplicative interaction (AMMI) analysis [19] was conducted to separate additive main effects from multiplicative interaction effects. (2) Genotype plus genotype-by-environment interaction (GGE) biplot analysis [31] was performed to visualize genotype–environment interactions and identify winning genotypes for specific environments. Both AMMI and GGE biplot analyses were conducted using R statistical software version 3.2.1 [64]. The AMMI model quantitatively assesses stability parameters, while GGE biplot analysis offers a graphical interpretation of genotype performance patterns across diverse environmental conditions [65].

## 5. Conclusions

Morkhor 60 demonstrated promising grain yield performance across two years, achieving the highest yields in Phu Khiao District, Chaiyaphum Province, during rainy seasons (1954–2330 kg/ha) and strong performance in Chum Phae District, Khon Kaen Province, during dry seasons (1294–1789 kg/ha). This complementary performance makes Morkhor 60 ideal for crop rotation between Phu Khiao and Chum Phae locations, optimizing yields across seasonal cycles. The variety outperformed CM 60 by 13% overall and showed generally promising results across both rotation environments, confirming its potential for integrated cropping systems.

## Figures and Tables

**Table 1:** Soil characteristics, geographic coordinates, and planting dates of thirteen test environments for soybean variety evaluation in Northeastern Thailand during 2022–2023.

Locations Code *	Seasons	Soil Texture	pH	Total N	Available P	Exchangeable K	Planting	Mean Grain Yield
				(%)	(mg/kg)	(mg/kg)	Date	(kg/ha)
KK-R-22	Rainy	Loamy Sand	6.28	0.01	40.83	25.03	29-Jul-2022	1366 c
CP-R-22	Rainy	Clay	7.14	0.11	14.56	121.00	30-Jul-2022	1123 de
PK-R-22	Rainy	Sandy loam	6.25	0.89	96.00	157.22	05-Jul-2022	1685 b
KK-D-22	Dry	Loamy Sand	5.57	0.03	56.30	69.99	08-Dec-2022	1984 a
CP-D-22-1	Dry	Clay	7.00	0.11	2.08	182.11	24-Dec-2022	1444 c
CP-D-22-2	Dry	Clay	7.13	0.11	18.00	135.25	24-Dec-2022	1272 cd
KK-R-23	Rainy	Loamy Sand	5.58	0.03	57.50	128.21	24-Jun-2023	1374 c
CP-R-23	Rainy	Loam	7.19	0.04	11.25	59.82	12-Aug-2023	1001 e
PK-R-23	Rainy	Sandy loam	6.23	0.97	87.50	113.03	07-Jul-2023	1979 a
KS-R-23	Rainy	Clay	7.11	0.47	5.50	830.03	12-Aug-2023	749 f
KK-D-23	Dry	Loamy Sand	6.58	0.01	36.52	44.00	10-Dec-2023	1776 b
CP-D-23	Dry	Clay	7.01	0.11	18.33	220.05	24-Dec-2023	1137 de
PK-D-23	Dry	Loam	5.52	0.02	81.41	137.78	25-Dec-2023	1025 e

* Location code: (KK-R-22) KKU, Khon Kaen Province in rainy season 2022; (CP-R-22) Chum Phae District, Khon Kaen Province in rainy season 2022; (PK-R-22) Phu Khiao District, Chaiyaphum Province in rainy season 2022; (KK-D-22) KKU, Khon Kaen Province in dry season 2022; (CP-D-22-1) Chum Phae District, Khon Kaen Province in dry season 2022; (CP-D-22-2) Chum Phae District, Khon Kaen Province in dry season 2022; (KK-R-23) KKU, Khon Kaen Prov-ince in rainy season 2023; (CP-R-23) Chum Phae District, Khon Kaen Province in rainy season 2023; (PK-R-23) Phu Khiao District, Chaiyaphum Province in rainy season 2023; (KS-R-23) Khok Sa-at District, Chaiyaphum Province in rainy season 2023; (KK-D-23) KKU, Khon Kaen Province in dry season 2023; (CP-D-23) Chum Phae District, Khon Kaen Province in dry season 2023; (PK-D-23) Phu Khiao District, Chaiyaphum Province in dry season 2023. Different letters following the means within a column indicate significant differences as determined by the least significant difference (LSD) test.

**Table 2:** Mean square values from combined ANOVA for yield components and grain yield of four soybean genotypes across thirteen environments showing genotype (G), environment (E), and genotype × environment (G × E) interaction effects.

Source of Variation	df	Number						100-Grain Weight	Grain Yield
		Nodes	Branches	Pods per Main Stem	Pods per Branch	Pods per Plant	Grains per Pod		
Envi. (E)	12	190.80 **	29.09 **	3306.90 **	12,618.00 **	26,456.00 **	0.46 **	27.89 **	2,374,800 **
Rep./E	39	0.66	0.43	13.71	90.30	123.70	0.01	0.52	64,616
Genotype (G)	3	6.06 **	37.70 **	322.00 **	7016.50 **	4336.50 **	0.76 **	58.31 **	717,184 **
G × E	36	3.45 **	2.29 **	61.14 **	1186.70 **	1448.50 **	0.28 **	2.31 **	142,382 **
error	117	0.23	0.25	5.55	43.40	47.10	0.01	0.31	12,079
CV (%)		3.64	12.10	8.67	22.62	12.2	4.04	4.02	7.97

CV = coefficient of variation; ** = significantly different at *p* < 0.01; Envi. = environment; Rep. = replication; df = degrees of freedom.

**Table 3:** Grain yield performance of four soybean genotypes across six environments during rainy and dry seasons in 2022, demonstrating the promising performance of the ‘Morkhor 60’ variety.

Varieties/Lines	The Grain Yield (kg/ha) in Rainy and Dry Seasons in 2022						Mean
	Rainy Season			Dry Season			
	KK-R-22	CP-R-22	PK-R-22	KK-D-22	CP-D-22-1	CP-D-22-2	
Morkhor 60	1428 AB	1213 B	1954 A	2046 A	1789 A	1302 B	1622
SJ 5	1237 BC	1456 A	1500 C	1910 B	1736 A	1644 A	1581
223*Lh-85	1161 C	856 C	1589 BC	1846 B	1162 B	1044 C	1276
CM 60	1637 A	969 C	1696 B	2135 A	1090 B	1099 C	1438
Mean	1366	1123	1685	1984	1444	1272	1479
F-test	**	**	**	**	**	**	
CV%	10.39	11.52	5.13	4.22	10.87	7.21	

CV = coefficient of variation; ** = significantly different at *p* < 0.01. Different letters following the means within a column indicate significant differences as determined by the least significant difference (LSD) test.

**Table 4:** Grain yield performance of four soybean genotypes across seven environments during rainy and dry seasons in 2023, confirming the consistent promise of the ‘Morkhor 60’ variety.

Varieties/Lines	The Grain Yield (kg/ha) in the Rainy and Dry Seasons of 2023							Mean
	Rainy Season				Dry Season			
	KK-R-23	CP-R-23	PK-R-23	KS-R-23	KK-D-23	CP-D-23	PK-D-23	
Morkhor 60	1434 A	1101 A	2330 A	818 A	1733 B	1294 A	979 B	1384
SJ 5	1320 B	1112 A	2190 A	851 A	1781 B	1077 BC	889 B	1317
223*Lh-85	1300 B	942 B	1596 B	672 B	1557 B	975 C	1208 A	1179
CM 60	1443 A	850 B	1798 B	656 B	2035 A	1201 AB	1024 B	1287
Mean	1374	1001	1979	749	1776	1137	1025	1308
F-test	**	**	**	**	**	**	**	
CV%	2.99	7.09	7.51	6.61	8.61	8.95	8.51	

CV = coefficient of variation; ** = significantly different at *p* < 0.01. Different letters following the means within a column indicate significant differences as determined by the least significant difference (LSD) test.

**Table 5:** Mean square values from combined ANOVA for protein, oil, fiber, and ash content of four soybean genotypes across seven environments showing genotype (G), environment (E), and genotype × environment (G × E) interaction effects.

Source of Variation	df	Oil (%)	Protein (%)	Source of Variation	df	Ash (%)	Fiber (%)
Envi. (E)	6	27.76 **	18.27 **	Envi. (E)	6	20.77 **	76.43 **
Rep./E	7	0.22	0.07	Rep./E	14	0.00	1.67
Genotype (G)	3	27.77 **	70.80 **	Genotype (G)	3	2.65 **	13.39 **
G × E	18	1.40 **	5.69 **	G × E	18	3.56 **	3.72 **
error	21	0.06	0.47	error	42	0.00	1.26
CV (%)	55	1.64	1.78	CV (%)	83	0.72	7.83

CV = coefficient of variation; ** = significantly different at *p* < 0.01; Envi. = environment; Rep. = replication; df = degrees of freedom.

**Table 6:** Chemical composition of four soybean genotypes across seven environments in Northeastern Thailand during 2022–2023.

Genotype	Ash (%)	Fiber (%)	Oil (%)	Protein (%)
Morkhor 60	5.53 C	14.70 A	14.66 C	39.63 B
SJ 5	5.63 B	15.29 A	13.38 D	40.74 A
223*Lh-85	5.66 B	13.84 B	15.72 B	35.53 D
CM 60	6.31 A	13.54 B	16.65 A	38.20 C
Mean	5.78	14.34	15.10	38.53
Environment (E)	**	**	**	**
Genotype (G)	**	**	**	**
E × G	**	**	**	**
CV (%)	0.72	7.83	1.64	1.78

CV = coefficient of variation; ** = significantly different at *p* < 0.01. Different letters following the means within a column indicate significant differences as determined by the least significant difference (LSD) test.

## Data Availability

The data presented in this study are available upon request from the corresponding author.
